# Unveiling the insecticidal efficiency of *Viola ignobilis* against *Macrosiphum rosae* and *Agonoscena pistaciae*: From chemical composition to cytotoxicity analysis

**DOI:** 10.1016/j.heliyon.2024.e40636

**Published:** 2024-11-22

**Authors:** Mohammad Sadegh Taghizadeh, Ali Niazi, Bernhard Retzl, Christian W. Gruber

**Affiliations:** a Institute of Biotechnology, School of Agriculture, Shiraz University, Shiraz, Iran; b Center for Physiology and Pharmacology, Medical University of Vienna, 1090, Vienna, Austria

**Keywords:** Cyclic peptide, GC-MS, MALDI-TOF MS, Plant extract, Small molecules, Violaceae

## Abstract

Currently, there is a growing preference for eco-friendly bioinsecticides over chemical insecticides due to their safety. Plant extracts have emerged as a promising solution for this purpose. Therefore, this study aimed to evaluate the insecticidal effectiveness of *Viola ignobilis* extract against two key pests of rose aphid (*Macrosiphum rosae*) and pistachio psylla (*Agonoscena pistaciae*). Significant compounds were identified using GC-MS and MALDI-TOF MS. Three bioassay methods were employed to assess the extract's insecticidal potential, and its cytotoxicity was tested on HEK293 cells. Results revealed that the highest insecticidal efficacy occurred at a concentration of 20 mg/mL after a 72 h exposure. The contact bioassay method displayed greater efficiency against *M. rosae* than *A. pistaciae*, while the oral bioassay demonstrated the highest efficiency against *A. pistaciae*. The extract also acted as a feeding deterrent, with indices of 77.47 ± 7.98 % and 87.98 ± 3.84 % for *A. pistaciae* and *M. rosae*, respectively. Furthermore, the insecticidal potency of the extract was assessed, resulting in LC_50_ values of 3.58 mg/mL and 6.77 mg/mL for the contact bioassay, and 0.87 mg/mL and 0.61 mg/mL for the oral bioassay against *M. rosae* and *A. pistaciae*, respectively. Importantly, the extract showed no detrimental cytotoxic effects on the HEK293 cell line within the tested concentration range, indicating its potential safety as a bioinsecticide. Overall, these findings highlight the potential of *V. ignobilis* extract as a promising candidate for further development in pest control.

## Highlights

• MALDI-TOF MS and GC-MS identified diverse bioactive compounds in the extract.
• Oral toxicity showed higher insecticidal potency of extract against both pests.
• Extract deterred the feeding of both pests confirming its insecticide potential.
• Extract showed no remarkable cytotoxic effect against control HEK293 up to 8 mg/mL.

## Graphical abstract

**Figure:**
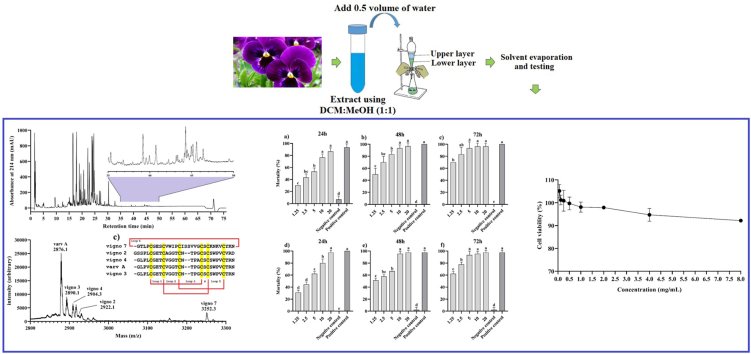

## 1 Introduction

The exposure of crops to pests has always been a challenge for farmers, with their primary focus being on protecting them. Therefore, it is the duty of the researchers to propose solutions that fight pests without having negative effects on the environment. Specifically, rose aphids (*Macrosiphum rosae*) and pistachio psylla (*Agonoscena pistaciae*) are key pests worldwide that pose a great threat to farmers. By sucking plant sap with their stylets, they cause curling leaves, deforming fruit, general wilting, and drying of the entire plant [1,2]. Additionally, they secrete honeydew on branches and leaves, resulting in the absorption of dust and providing a good medium for mold growth. The origin of *A. pistaciae* and *M. rosae* has been reported for the first time from Iran and the west of the Palearctic region, respectively [2,3], but now they are spread all over the world.

Farmers consider the chemical method to be the most common way of fighting these pests, particularly in developing countries. However, the continuous use of chemical insecticides has consequences such as long-lasting environmental presence, pest resistance, and irreparable damage to the ecosystem [4,5]. Consequently, the use of bioinsecticides has gained attention from researchers, and currently working on their development [6]. Bioinsecticides contain of ingredients derived from living organisms and can include microorganisms, plant extracts, and biomolecules [7]. Bioinsecticides based on plant compounds offer several advantages, including safety for humans and animals, strong pest control capabilities, and eco-friendliness [8].

The merit of plants as a rich source of bioactive compounds has been confirmed through various research studies in traditional medicinal and agricultural applications [[9], [10], [11], [12]]. The potential of plant compounds to become bioinsecticides has been established due to their suggested role in the plant's defense system [7,13]. Among the important peptides in plants, cyclotides exhibit various intrinsic bioactivities, including protease inhibition [14], immunosuppression [15], antibacterial [16], cytotoxicity [17], as well as nematocidal [18], molluscicidal [19], and insecticidal [20] activities. Additionally, plant secondary metabolites possess significant biological activities, including anticancer, antioxidant, and antimicrobial [[21], [22], [23]], making plants a diverse source of bioactive compounds that have benefited humans throughout history. Hence, research on plant bioactive compounds remains an emerging topic despite extensive studies in the field of drug discovery. Recently, a commercially produced bioinsecticide named Sero-X, based on the extract of *Clitoria ternatea*, a cyclotide-bearing plant, has been developed. Sero-X acts against a wide range of cotton pests without adverse effects on humans and animals [24]. Furthermore, the ability of plant compounds to control livestock pests, such as nematodes and helminths, has been proven [25,26]. Thus, plant compounds are fantastic molecules with potential use as bioinsecticides in the agricultural industry.

*Viola ignobilis*, a medicinal plant from the Violaceae family native to Asia, is renowned for its natural phytoconstituents with potential applications in modern medicine for various health conditions. This ethnobotanical herb of India plays a significant role in the Ayurvedic System of Medicine, showcasing antibacterial, antioxidant, and anti-inflammatory properties [27,28]. Research on *V. ignobilis* has primarily focused on identifying and understanding the pharmacological properties of cyclotides. Various cyclotides, such as varv A, vigno 1–10, kalata B1, cycloviolacin O2, and cycloviolacin O9, have been previously identified from *V. ignobilis* [29,30]. A notable study highlighted the anticancer potential of cyclotides extracted from *V. ignobilis* [31]. In traditional medicine, the plant's aerial parts are utilized in treating bronchitis, cough, asthma, and nervous disorders. Despite these known attributes, the chemical composition and insecticidal properties of *V. ignobilis* still lack comprehensive understanding. In this regard, the current study was fulfilled to demonstrate the efficacy of an extract as a bioinsecticide targeting two of the most important foliar sap-sucking pests of pistachio (*A. pistaciae*) and rose (*M. rosae*) plants. To achieve this, the extract of *Viola ignobilis* was tested for insecticidal activity in three different manners to obtain quantitative concentration response data, such as LC_50_. The activity of the extract was compared to that of Chlorpyrifos, a synthetic pesticide used as a positive control. Additionally, the bioactive compounds in the extract were identified using MALDI-TOF MS and GC-MS, and the cytotoxicity of the extract was assessed against the HEK293 human normal cell line. The findings of our study demonstrated the potential of developing this extract as a bioinsecticide for controlling *M. rosae* (Hemiptera: Aphididae) and *A. pistaciae* (Hemiptera: Psyllidae).

## 2 Materials and methods

### 2.1 Chemicals and reagents

All chemical compounds were procured in their standard forms from Merck Company. The α-Cyano-4-hydroxycinnamic acid (CHCA) matrix was sourced from Sigma-Aldrich®. Commercial formulation of Chlorpyrifos (Bayer CropScience) was acquired from the local market in Shiraz, Iran. Cell culture supplies such as DMEM culture medium, fetal bovine serum (FBS), MTT reagent, penicillin, and streptomycin were obtained from Dena-Zist Company, Iran. HEK293 cell lines were generously gifted by Dr. Amin Ramezani from the Cancer Research Center, Shiraz University of Medical Sciences, Iran.

### 2.2 Plant materials

The *V. ignobilis* plant in its flowering stage was acquired from a local market in Shiraz, Iran. The plant material was authenticated by a qualified botanist at the herbarium of Shiraz University (Shiraz, Iran), and a voucher specimen (V12543) was obtained. The plant materials were cleaned, and subsequently, the aerial parts, including leaves and flowers, were dried at room temperature under shade.

### 2.3 Plant extraction

The dried plant material (100 g) was powdered using liquid nitrogen and subjected to extraction using a mixture of dichloromethane and methanol (1:1 (v/v)) in a ratio of 1:10 (w/v) [32]. The mixture was continuously shaken at 25 °C for 18–24 h. After filtration, deionized water (ddH_2_O) was added up to half of the final volume and then the water/methanol phase was collected. The solvent was evaporated using a rotary evaporator, and the resulting extract was lyophilized. The extraction yield was determined by dividing the weight of the extract powder by the weight of the plant material, expressed as a percentage.

### 2.4 Analysis of plant extract

#### 2.4.1 MALDI-TOF MS analysis

Considering the *V. ignobilis* is a cyclotide-containing plant, the cyclotide profile of the extract was evaluated using matrix-assisted laser desorption ionization time-of-flight mass spectrometry (MALDI-TOF MS 4800 Analyser, AB Sciex, Framingham, MA, United States). For this analysis, α-cyano-4-hydroxycinnamic acid (CHCA) was used as the matrix, which was prepared in a solution consisting of 50 % acetonitrile (ACN) and 0.1 % trifluoroacetic acid (TFA). A 0.5 μL of the dissolved extract in ddH_2_O was mixed with 3 μL of the matrix solution and 1 μL of the mixture was directly spotted onto the MALDI target plate. The analysis was conducted in reflector positive ion mode, acquiring a total of 2500–5000 shots per spectrum. The laser intensity was set within the range of 3200–3800. The spectra were obtained, processed, and analyzed using the Data Explorer Software from AB Sciex. Possible cyclotides within the extract were recognized by comparing their monoisotopic molecular weights with those documented for *V. ignobilis* species in the CyBase database [33].

#### 2.4.2 GC-MS analysis

To detect the volatile secondary metabolites, GC-MS analysis was carried out using a constant flow of helium at a rate of 1 mL/min as the carrier gas [34]. The column temperature was set at an initial value of 50 °C and subsequently raised at a rate of 5 °C per minute until it reached 230 °C. It was then maintained at this temperature for a duration of 2 min. The temperature was subsequently raised to 290 °C at a rate of 30 °C/min and maintained at this temperature for 3 min. The temperature of injector and mass spectrometry (MS) transmission lines were set at 250 °C and 260 °C, respectively. Afterward, a 1 μL aliquot of the sample was injected into the instrument, and analysis was performed in electron ionization (EI) mass spectra with an ionization voltage of 70 eV, encompassing a range of 400–1000 *m*/*z*. Additionally, the temperature of ion source was adjusted to 200 °C. Finally, the compounds were compared and identified based on their retention time and mass spectra, using the NIST 11 database as a reference.

#### 2.4.3 RP-HPLC analysis

Additionally, the compound profile of the extract was examined using analytical RP-HPLC equipped with a Kromasil C_18_ column (dichrom GmbH, Marl, Germany; 250 × 4.6 mm, 5 μm, 100 Å) at a flow rate of 1 mL/min. A gradient of 5–65 % (1 % per minute) solvent B, consisting of 90 % ACN, 9.9 % deionized water (ddH_2_O), and 0.1 % TFA (v/v/v), was used according to the previously described study [32].

### 2.5 Insect rearing

The mature insects of *M. rosae* (Hemiptera: Aphididae) and *A. pistaciae* (Hemiptera: Psyllidae) were gathered from the Botanical Garden of Shiraz University, Shiraz, Iran, and the pistachio fields in Rafsanjan, Iran, respectively. We specifically chose plants that had not been treated with any pesticides for the past three years to collect the insects. Subsequently, the insects were transported to the laboratory and individually reared on their respective host plants under controlled conditions. The rearing conditions included a temperature of 25 ± 2 °C, a photoperiod of 16:8 (light:dark), and a relative humidity of 55 ± 5 %. For all bioassay experiments, we used 4th-instar nymphs of *M. rosae* and 5th-instar nymphs of *A. pistaciae*.

### 2.6 Bioassay evaluation

The extract was assessed as an insecticidal agent against two foliar sap-sucking pests using three different procedures: contact toxicity, oral bioassay, and determination of deterrent activity. The experiments were conducted separately, and data recording was conducted using a microscope after exposure times of 24, 48, and 72 h. For this purpose, under the microscope, the insects were gently touched with a camel brush and were considered alive if they moved in response, and dead otherwise.

#### 2.6.1 Contact toxicity

In this bioassay, the nymphs were placed in a nematode strainer with a mesh size of 300 μm and then dipped into different concentrations (2.5, 5, 10, and 20 mg/mL) of the extract for 10 s. Afterward, the nymphs were carefully collected using a camel brush and transferred onto leaf discs of their respective host plants in Petri dishes. The Petri dishes were designed with a wet cotton layer at the bottom to maintain moisture and a net cloth-covered hole (2 cm in diameter) at the top for ventilation, following the method described by Ahmed et al. [35]. Additionally, a filter paper was placed on top of the cotton layer, on which the leaf discs were positioned. A total of fifteen nymphs were introduced to each leaf disc, after which the Petri dishes were placed in a controlled environment room for incubation. For the controls, positive control was prepared using the concentration of Chlorpyrifos specified by the manufacturer (0.612 mg/mL), while the negative control consisted of water. Mortality data was recorded by assessing the viability of the insects through needle stimulation. The percentage of mortality was determined using the equation provided by Abbott [36].

#### 2.6.2 Oral bioassay

The oral bioassay was conducted following the method previously described by Sadeghi et al. [37], with some alternations. Briefly, the feeding apparatus was assembled as described in the reference mentioned above. Subsequently, 200 μL of artificial diet (20 % sucrose) containing various concentrations of the extract, including 1.25, 2.5, 5, 10, and 20 mg/mL, was pipetted between two Parafilm membranes, and the nymphs were transferred onto the sachet using a camel brush. The apparatus was then placed upside down in the wells and incubated in a room under controlled conditions. The sachets were replaced every two days. As negative and positive controls, water and Chlorpyrifos (0.612 mg/mL) were used in the artificial diet, respectively. Mortality data and the corresponding percentage of mortality were analyzed as described above.

#### 2.6.3 Determination of deterrent activity

The deterrent activity of the extract was assessed using the method provided by Omer et al. [38]. Two leaf discs from each host plant were prepared separately and immersed in the extract solution (20 mg/mL) as the treatment and water as the control for a duration of 2 min. Subsequently, the leaf discs were positioned in opposing directions in a Petri dish, with filter paper placed on top of moist cotton. Fifteen nymphs of each insect were transferred to the center point between the two leaf discs. The Petri dishes were sealed and subsequently incubated for the specified duration in a room with controlled conditions. Finally, the number of insects on the leaf discs was independently counted, and the deterrent index was calculated using the equation provided by Pascual-Villalobos and Robledo [39]: Deterrent index = (N_C_ – N_T_)/(N_C_ + N_T_) × 100, where N_C_ represents the number of insects on the control leaf discs and N_T_ represents the number of insects on the treated leaf discs.

### 2.7 Cytotoxicity assay

The MTT assay method was employed to assess the cytotoxicity activity of the extract against the human embryonic kidney cell line (HEK293), following the procedure described in a previous study [40]. The DMEM medium containing 10 % fetal bovine serum, 100 μg/mL streptomycin, and 100 U/mL penicillin, was utilized for seeding the cell line. The cells were kept in a humidified incubator at a temperature of 37 °C with a 5 % CO_2_ atmosphere. After sequential passages, cells were seeded at a concentration of 1 × 10^4^ cells per well in a 96-well plate and incubated overnight under the aforementioned conditions. The medium was substituted with different concentrations of the extract (0.0625, 0.125, 0.25, 0.5, 1, 2, 4, and 8 mg/mL), and the plate was incubated under the same conditions for 24 h. Subsequently, the treatments were discarded, and the cells were rinsed with ice-cold PBS buffer (pH 7.2). MTT reagent (0.5 mg/mL, prepared in medium) was added to each well, and the plate was incubated for 4 h under the same conditions. Then, the medium was discarded, and 100 μL of DMSO was introduced to each well, followed by incubation for 15 min with gentle shaking. The optical absorbance at 570 nm was quantified using a microplate reader (Bio-Rad, Richmond, CA, USA). Positive and negative controls were included using Triton X-100 and fresh medium, respectively. Cell viability was determined using the following formula: (T_24_ - C^+^)/(C^−^ - C^+^) × 100, where T_24_, C^+^, and C^−^ denote the absorbance of the sample, positive control, and negative control, respectively.

### 2.8 Data analysis

All insecticide experiments were conducted in three biological and five technical replicates, with each replicate consisting of fifteen nymphs. The data were subjected to analyze using non-linear regression (curve fit), and the mortality percentage was assessed based on the concentration-response curve using the GraphPad Prism 8.0 software (GraphPad Software Inc., San Diego, CA). The medium lethal concentration (LC_50_) and its corresponding 95 % confidence interval (95 % CI) were estimated in the GraphPad Prism software, following the method described in another study [41]. The accuracy of the analyzed data was also verified by calculating the *R*^*2*^ value.

## 3 Results

### 3.1 Preparation of extract

**Fig. 1:**
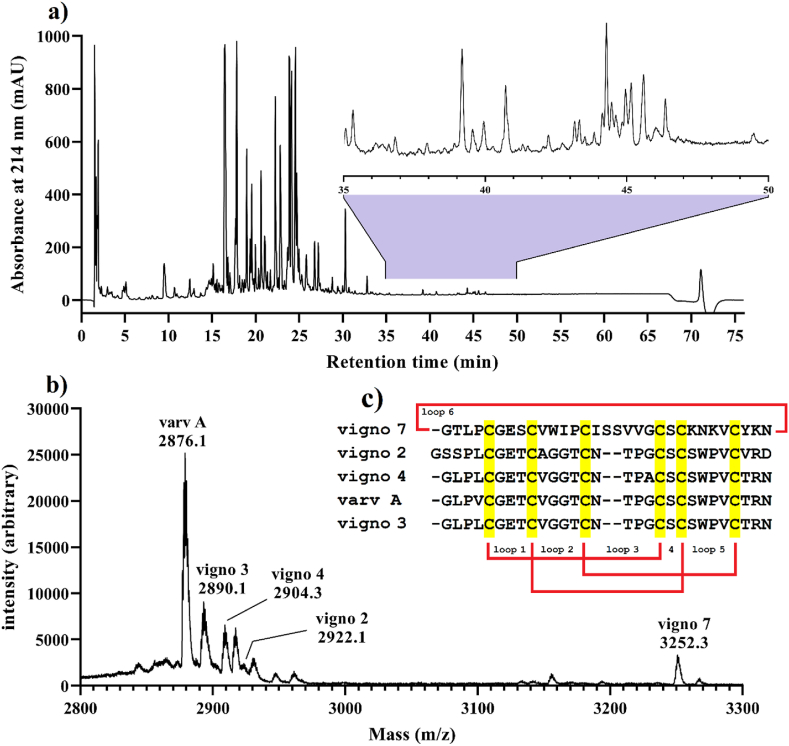
Peptide profile analysis of *V. ignobilis* extract. a) Analytical RP-HPLC analysis of extract with the magnification of the cyclotide eluting region (inset), b) MALDI-TOF MS analysis of extract, labeling by the common name and related monoisotopic molecular weight of their possible counterparts reported from *V. ignobilis* in the CyBase database [33]. c) Sequence alignment and cysteine knot connectivity of identified cyclotides in the extract of *V. ignobilis*.

Table 1 presents the amino acid sequences, the experimental and theoretical mass (Da), and mass deviation (ppm) of different cyclotides identified from *V. ignobilis* extract. Since cyclotides are typically eluted towards the end of the C18 chromatographic run and have a molecular weight ranging from 2700 to 3500 *m*/*z* [32], their presence in the extract was confirmed using RP-HPLC (Fig. 1a) and MALDI-TOF MS (Fig. 1b). By comparing the monoisotopic molecular weight of cyclotides obtained from MALDI-TOF MS analysis with those documented for *V. ignobilis* species in the CyBase database, the putative cyclotides detected in the extract were varv A (monoisotopic molecular weight: 2876.1 Da), vigno 3 (2890.1 Da), vigno 4 (2904.3 Da), vigno 2 (2922.1 Da), and vigno 7 (3252.3 Da). Additionally, the sequence alignment of identified cyclotides is illustrated in Fig. 1c.

**Table 1:**
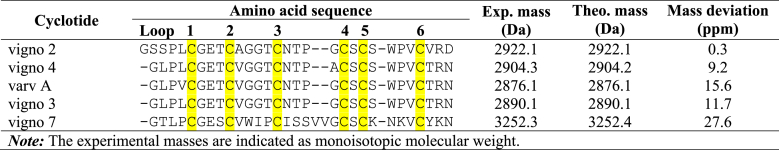
The amino acid sequences, experimental and theoretical mass (Da), and mass deviation (ppm) of cyclotides identified from *V. ignobilis* extract.

On the other hand, GC-MS analysis detected the presence of various small molecules in the extract, including 1-nitro-2-propanol (Molecular weight: 105.43 g/mol), methyl salicylate (152.047 g/mol), 6,6-dideutero-nonen-1-ol-3 (144.148 g/mol), 4,4,5,8-tetramethylchroman-2-ol (206.131 g/mol), (E)-Stilbene (180.094 g/mol), phenanthrene (178.078 g/mol), methyl palmitate (270.256 g/mol), 4-cyclopentene-1,3-dione, 4,5-bis(4-fluorophenyl)-2-phenyl- (360.096 g/mol), diisooctyl phthalate (390.277 g/mol), and bis(2-ethylhexyl) phthalate (390.277 g/mol) (Table 2). Based on their peak area, the most abundant small molecules found in the extract were 6,6-dideutero-nonen-1-ol-3 (11.3 %), 4-cyclopentene-1,3-dione, 4,5-bis(4-fluorophenyl)-2-phenyl- (12.56 %), and bis(2-ethylhexyl) phthalate (60.9 %). Subsequently, the extract was tested to determine its insecticidal activity against two major foliar sap-sucking pests of pistachio and rose plants.

**Table 2:** Chemical compositions of *V. ignobilis* extract identified using GC-MS analysis.

Name of compound	RT (min)	Area (%)	Pesticidal activity	Reference
1-nitro-2-propanol	3.32	3.37	Not available	–
methyl salicylate	14.83	5.24	**Yes** (insecticide)	[53]
6,6-dideutero-nonen-1-ol-3	22.84	11.3	Not available	–
4,4,5,8-tetramethylchroman-2-ol	26.8	1.77	Not available	–
(E)-stilbene	28.19	1.67	**Yes** (insecticide)	[54]
phenanthrene	29.94	2.11	**Yes** (insecticide)	[55]
methyl palmitate	32.04	0.68	**Yes** (acaricide)	[56]
4-cyclopentene-1,3-dione, 4,5-bis(4-fluorophenyl)-2-phenyl-	37.58	12.56	Not available	–
diisooctyl phthalate	41.15	0.4	Not available	–
bis(2-ethylhexyl) phthalate	41.52	60.9	**Yes** (insecticide)	[57]
Total identified		100		

### 3.2 Extraction yield

In this study, the extraction yield of the extract obtained from methanol/water phase was determined to be 10.2 ± 0.84 % of the plant’s dry weight. The resulting extract was appeared as purple-colored solid powder after freeze-drying.

### 3.3 Contact toxicity bioassay

**Fig. 2:**
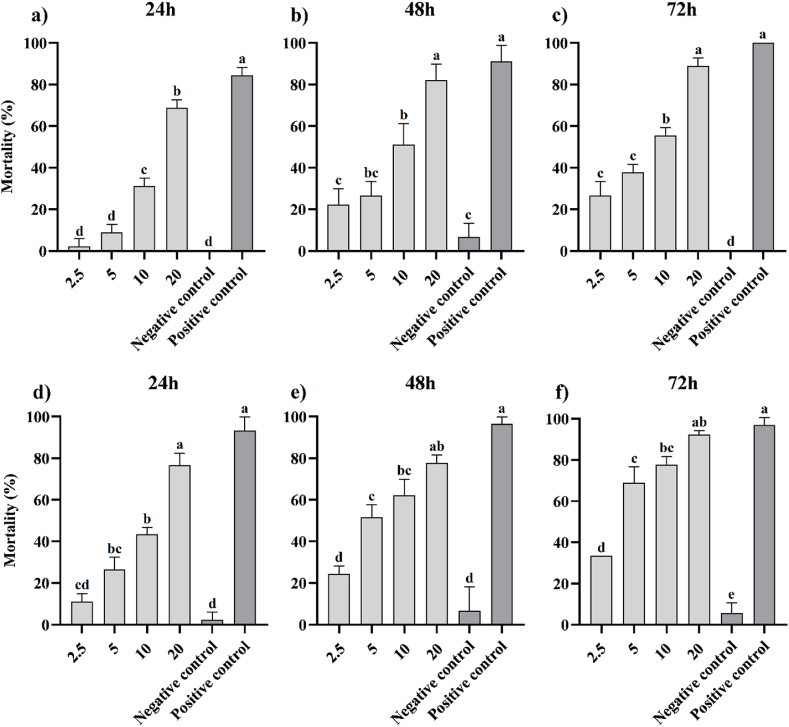
Contact toxicity bioassay of extract prepared from *V. ignobilis* against *A. pistaciae* (a–c) and *M. rosae* (d–f) at different concentrations and exposure times. Concentrations were expressed as mg extract per mL. All experiments were performed in three biological and five technical replicates. Data were shown as mean ± SD (standard deviation). Chlorpyrifos (0.612 mg/mL) and water were used as positive and negative controls, respectively. Also, a-e letters indicate the mean comparison analysis using Tukey test at *P-value* ≤ 0.01.

A contact toxicity bioassay was conducted using an extract derived from *V. ignobilis* against *A. pistaciae* and *M. rosae*. The bioassay, conducted at 24 h (Fig. 2a), 48 h (Figs. 2b), and 72 h (Fig. 2c) against *A. pistaciae*, and at 24 h (Figs. 2d), 48 h (Fig. 2e), and 72 h (Fig. 2f) against *M. rosae*, revealed an increase in mortality rates with increasing concentration and exposure duration for both pests (Fig. 2). According to the results, the highest mortality was obtained at 20 mg/mL of the extract after 72 h of exposure for both pests (92.22 ± 1.92 % for *M. rosae* and 88.89 ± 3.14 % for *A. pistaciae*). Generally, the insecticidal efficacy of the extract against *M. rosae* was more effective than against *A. pistaciae*. When compared to the positive control, Chlorpyrifos exhibited the highest mortality against both pests (84.45 ± 3.14 %, 91.11 ± 6.28 %, and 100 ± 0.0 % after 24 h, 48 h, and 72 h of exposure for *A. pistaciae*; 93.33 ± 6.66 %, 96.44 ± 3.36 %, and 97.11 ± 3.42 % after 24 h, 48 h, and 72 h of exposure for *M. rosae*, respectively). Furthermore, the lowest mortality was detected in the negative control for both pests (ranging from 0.0 ± 0.0 % to 6.67 ± 5.44 % against *A. pistaciae* and from 2.22 ± 3.85 % to 6.67 ± 11.54 % for *M. rosae*), which can be attributed to natural deaths. The statistical analysis indicated a significant model fitness at a *P-value* < 0.01 for the mortality percentage caused by contact toxicity at different concentrations and times in both pests. Overall, the maximum mortality percentage of the extract against *M. rosae* and *A. pistaciae* at 20 mg/mL after 72 h of exposure respectively was 5.03 % and 11.11 % lower than the positive control, but there was no significant difference between them at a *P-value* < 0.01. In the contact toxicity of *A. pistaciae*, the variability in susceptibility decreased at 24 h of exposure and increased at 48 and 72 h of exposure. In contrast, the variability in susceptibility of *M. rosae* decreased at 24 h of exposure, then increased at 48 h of exposure, and finally decreased again at 72 h of exposure.

By calculating the LC_50_ value, the contact toxicity potency of the extract increased with exposure time (Table 3). The LC_50_ value of the extract against *A. pistaciae* was estimated at 14.13, 8.68, and 6.77 mg/mL after 24, 48, and 72 h of exposure, respectively. Similarly, the LC_50_ value against *M. rosae* was estimated at 10.45, 5.85, and 3.58 mg/mL after 24, 48, and 72 h of exposure, respectively. Comparatively, the contact toxicity potency of the extract against *M. rosae* was higher than against *A. pistaciae*. Additionally, the slope, 95 % confidence interval, and *R*^*2*^ values for the mortality response of the extract in contact toxicity are summarized in Table 3.

**Table 3:** Contact bioassay of extract of *V. ignobilis* against *A. pistaciae* and *M. rosae* at different concentrations and exposure times.

Pest	Time (h)	LC_50_ (mg/mL)	95 % C.I.		Slope ±SD	*R*^*2*^
			Lower	Upper		
*A. pistaciae*	24	14.13	13.27	15.06	1.96 ± 0.46	0.98
	48	8.68	6.96	10.85	0.97 ± 0.77	0.87
	72	6.77	5.54	8.20	0.97 ± 0.54	0.91
*M. rosae*	24	10.45	9.23	11.87	1.58 ± 0.47	0.95
	48	5.85	4.85	7.01	1.01 ± 0.40	0.91
	72	3.58	3.03	4.14	1.56 ± 0.59	0.93

***Note:*** All experiments were done in three biological and five technical repeats. C.I., confidence interval; SD, standard deviation. *R*^*2*^, R squared.

### 3.4 Oral bioassay

**Fig. 3:**
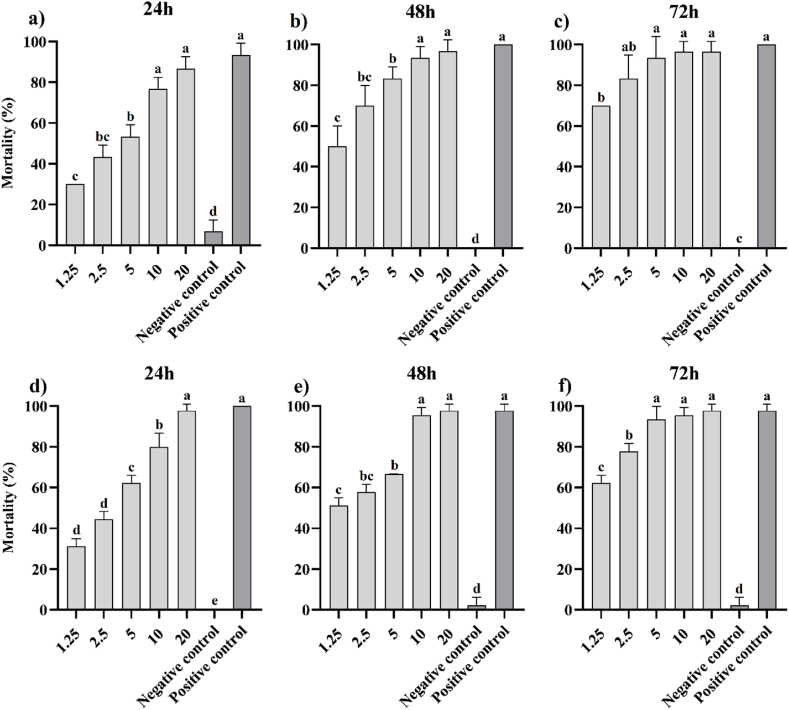
Oral bioassay of extract prepared from *V. ignobilis* against *A. pistaciae* (a–c) and *M. rosae* (d–f) at different concentrations and exposure times. Concentrations were expressed as mg extract per mL. All experiments were performed in three biological and five technical replicates. Data were shown as mean ± SD (standard deviation). Chlorpyrifos (0.612 mg/mL) and water were used as positive and negative controls, respectively. Also, a-d letters indicate the mean comparison analysis using Tukey test at *P-value* ≤ 0.01.

Considering that insecticides can be absorbed by plants and then enter the bodies of sucking insects through plant sap [42], conducting an oral bioassay can enhance our comprehension of their efficacy at biochemical levels. In an oral bioassay, an extract derived from *V. ignobilis* was tested against *A. pistaciae* and *M. rosae*. The results, observed after 24 h (Fig. 3a), 48 h (Figs. 3b), and 72 h (Fig. 3c) against *A. pistaciae*, and after 24 h (Figs. 3d), 48 h (Fig. 3e), and 72 h (Fig. 3f) against *M. rosae*, indicated that this method was more effective in inducing higher mortality rates against both pests compared to the contact bioassay method (Fig. 3). Accordingly, the results of the oral bioassay revealed that even low concentrations of the extract could cause mortality of more than 30 % in both pests after 24 h of exposure. Moreover, the mortality increased with higher concentrations and longer exposure times. The highest mortality was obtained at 20 mg/mL of the extract after 72 h of exposure for both pests (98.11 ± 3.14 % for *M. rosae* and 98.67 ± 1.88 % for *A. pistaciae*), which approached the efficacy of the positive control (Fig. 3). The insecticidal efficacy of the positive control was estimated at 100 ± 0.0 %, 97.78 ± 3.01 %, and 97.78 ± 3.14 % against *M. rosae*, and 93.33 ± 4.71 %, 100 ± 0.0 %, and 100 ± 0.0 % against *A. pistaciae* after 24, 48, and 72 h of exposure, respectively. On the other hand, the mortality caused by the negative control was 0.0 ± 0.0 %, 2.22 ± 3.11 %, and 2.22 ± 3.14 % against *M. rosae*, and 6.67 ± 4.71 %, 0.0 ± 0.0 %, and 0.0 ± 0.0 % against *A. pistaciae* after the respective exposure times. The statistical analysis revealed a significant level at a *P-value* < 0.01 for the percentage of mortality caused by the oral bioassay at different concentrations and times in both pests. The insecticidal efficacy of the extract at 20 mg/mL after 72 h of exposure against *M. rosae* was 0.34 % higher, while against *A. pistaciae* it was 1.33 % lower compared to the positive control, but there was no significant difference between them at a *P-value* < 0.01. Furthermore, in the oral bioassay of *A. pistaciae*, the variability in susceptibility increased at 24 h of exposure, followed by a decrease at 48 and 72 h of exposure. In contrast, the variability in susceptibility of *M. rosae* was similar to its contact toxicity.

Table 4 presents the LC_50_, slope, 95 % confidence interval, and *R*^*2*^ values obtained from the data analysis of the oral bioassay, highlighting the high sensitivity of both pests to the extract. The findings indicated that the potency of the extract against the two pests increased with higher concentrations and longer exposure times. Consequently, the LC_50_ value of the extract was calculated as 3.39, 1.25, and 0.61 mg/mL against *A. pistaciae*, and 2.87, 1.50, and 0.87 mg/mL against *M. rosae* after 24, 48, and 72 h of exposure, respectively (Table 4). Upon comparison, the potency of the extract in the oral bioassay against *A. pistaciae* was found to be higher than against *M. rosae*.

**Table 4:** Oral bioassay of extract of *V. ignobilis* against *A. pistaciae* and *M. rosae* at different concentrations and exposure times.

Pest	Time (h)	LC_50_ (mg/mL)	95 % C.I.		Slope ±SD	*R*^*2*^
			Lower	Upper		
*A. pistaciae*	24	3.39	2.86	3.99	0.96 ± 0.18	0.93
	48	1.25	0.90	1.55	1.21 ± 0.39	0.87
	72	0.61	0.18	0.95	1.17 ± 0.75	0.70
*M. rosae*	24	2.87	2.45	3.33	1.17 ± 0.22	0.95
	48	1.50	0.99	1.99	0.98 ± 0.31	0.84
	72	0.87	0.65	1.06	1.31 ± 0.36	0.92

***Note:*** All experiments were done in three biological and five technical repeats. C.I., confidence interval; SD, standard deviation. *R*^*2*^, R squared.

### 3.5 Deterrent activity

The deterrent activity was conducted to examine the impact of the extract on the behavior of the pests. The results indicated that the extract successfully deterred the feeding of both *A. pistaciae* and *M. rosae*, with deterrent indices (DI) of 77.47 ± 7.98 % and 87.98 ± 3.84 %, respectively (Table 5). When compared to the positive control, where the leaves treated with Chlorpyrifos (0.612 mg/mL) were used, *A. pistaciae* and *M. rosae* pests actively avoided feeding on them, resulting in DI values of 97.78 ± 3.85 % and 100 ± 0.0 %, respectively. These findings demonstrate that most of the pests preferred untreated leaves for feeding.

**Table 5:** Deterrent activity of extract of *V. ignobilis* against *A. pistaciae* and *M. rosae* at 20 mg/mL after 24 h.

Insect	Number of insects per leaves		DI (%)	Number of insects per leaves		DI (%)
	Treatment	Negative control		Positive control	Negative control	
*A. pistaciae*	1.11 ± 0.38	8.78 ± 0.51	77.47 ± 7.98	0.11 ± 0.19	9.89 ± 0.19	97.78 ± 3.85
*M. rosae*	0.78 ± 0.19	10.56 ± 0.38	87.98 ± 3.84	0.0 ± 0.0	11.0 ± 0.67	100 ± 0.0

***Note:*** Experiments were done in three biological and five technical repeats, and data were expressed as mean ± SD. DI: deterrent index. Water and chlorpyrifos were used as negative and positive controls, respectively.

### 3.6 Cytotoxicity activity

**Fig. 4:**
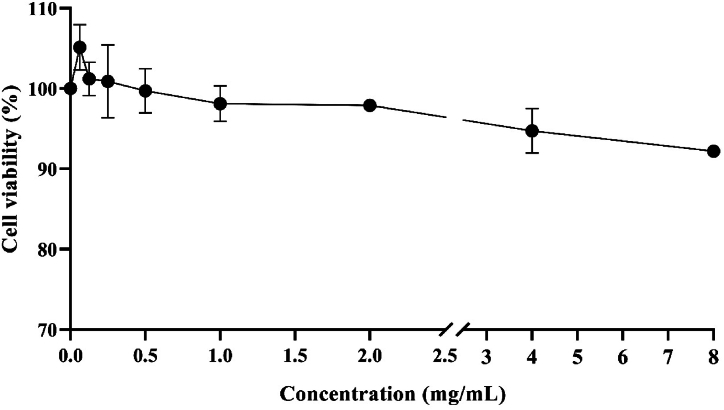
Cytotoxicity assay of *V. ignobilis* extract against HEK293 human cell line using MTT assay method. The experiment was performed in three biological and two technical replicates. Data were shown as mean ± SD (standard deviation).

To evaluate the effect of *V. ignobilis* extract on the HEK293 human control cell line, various concentrations were tested, chosen based on the oral bioassay conducted against pests in this study. No detrimental effects on the cells were detected; at the highest concentration, >92 % of cells were intact (7.81 % cell death) (Fig. 4). The cytotoxic activity of the identified compounds in the *V. ignobilis* extract has not been previously documented against HEK293 cells. Nevertheless, according to the analytical findings, even at concentrations of up to 8 mg/mL, the combination of these diverse compounds did not exhibit any major cytotoxic effects on HEK293 cells tested in this study. Therefore, this result provides confidence to explore this plant extract further, as it demonstrates its potential as a safe insecticide in the future.

## 4 Discussion

The emergence of resistance in *A. pistaciae* and *M. rosae* to synthetic insecticides poses a significant challenge in environmental preservation [43,44]. Consequently, these insecticides have been used extensively, causing irreparable damage to the environment and human health. As an alternative approach, the utilization of nature-derived compounds has gained attention. It has been demonstrated that some plant extracts, including *Clitoria ternatea*, *Bidens pilosa*, *Lantana camara*, *Lippia javanica*, *Tephrosia vogelii*, *Tithonia diversifolia*, and *Vernonia amygdalina*, have no adverse effects on beneficial insects [45,46]. Previous studies have investigated the efficacy of individual cyclotides and various plant extracts containing various secondary metabolites against different insects, revealing a range of insecticidal potencies [5,46]. Hence, we aimed to open new avenues for developing environmentally safe insecticides by finding new insecticidal plant extracts.

The results showed that the extract exhibited greater insecticidal potency and efficacy against *M. rosae* in the contact toxicity assay, with a mortality rate of 97.11 ± 3.14 % at 20 mg/mL and an exposure time of 72 h (LC_50_ = 3.58 mg/mL). On the other hand, it was more effective against *A. pistaciae* in the oral bioassay, with a mortality rate of 98.67 ± 1.88 % at 20 mg/mL and an exposure time of 72 h (LC_50_ = 0.61 mg/mL). This differential response may be due to unique physiological characteristics, metabolic pathways, or target sites of *A. pistaciae* that make it more vulnerable to the insecticidal properties of the extract, leading to higher mortality rates and lower LC_50_ values in comparison to *M. rosae*. Additionally, oral consumption of the extract by *A. pistaciae* may facilitate the availability of compounds for specific binding and inhibition of insect targets. The slope of the dose-response line indicates the change rate of mortality relative to the change rate of concentration [47]. A steep slope suggests low variability in insect susceptibility to a pesticide [47]. The steep slope observed in both pests and the significant difference between low and high concentrations suggest that the pest populations were homogeneous in susceptibility. This implies that even a relatively small increase in the concentration of the extract can lead to a higher mortality rate.

Furthermore, the extract acted as a deterrent, preventing both pests from feeding on the host leaves, with a deterrent index (DI) of more than 75 %. This demonstrates the antixenosis resistance mechanism in plants, where the extract negatively affects pest behavior [48]. A research has demonstrated that *V. ignobilis* extract prepared by maceration contains various cyclotides at concentrations ranging from 83.86 to 249.71 μg per gram of dried plant [29]. These cyclotides have demonstrated insecticidal activity at different concentrations in previous *in vitro* experiments. For instance, kalata B1, kalata B2, psyleio A, and cter M exhibited insecticidal activity against *H. armigera* at concentrations ranging from 0.5 to 1 μmol/g [24,49]. Besides, Oguis, Gilding, Huang, Poth, Jackson and Craik [49] showed that there is no significant difference in the insecticidal potency of *Clitoria ternatea* extract with or without cter M (The cyclotide with the highest abundance in *C. ternatea*). The ethanolic extract from *Hybanthus parviflorus*, another cyclotide-bearing plant, at a concentration of 1000 ppm, showed 100 % mortality against *Ceratitis capitata* larvae [50]. Additionally, kalata B1 negatively affected the development and survival of *H. punctigera* larvae at a concentration of 0.825 μmol/g, resulting in a 50 % mortality rate following 16 days of feeding [20]. Despite the modest levels of cyclotides present in our extract, these small amounts are capable of providing insecticidal effects according to previous studies. For instance, feeding *Diatraea saccharalis* larvae by a diet containing paragidin-br1 cyclotide significantly decreased their weight and size compared to the control, resulting in a mortality rate of 60 % [51]. Furthermore, cycloviolacins isolated from *Viola* spp. have recently been shown to have a deterrent impact on the feeding and probing behavior of *M. persicae* in a concentration-dependent manner [48]. To date, there is no documented evidence confirming the insecticidal attributes of the cyclotides identified in this study. However, given the nature of cyclotides in providing insecticidal properties, the cyclotides identified in this research may contribute to the insecticidal characteristics of the *V*. *ignobilis* extract. Conversely, drawing from prior research on the lethal low concentrations of cyclotides against insects, despite the relatively low concentration of cyclotides within the *V*. *ignobilis* extract, they may still exhibit a small part of insecticidal activity, either independently or through synergistic effects with other compounds present in the extract [52]. This finding aligns with the bioinsecticide Sero-X, which is a cyclotide-containing extract from *C. ternatea* [24]. Therefore, future studies focused on purifying and evaluating the insecticidal properties of these cyclotides have the potential to dispel these uncertainties.

Moreover, the extract contains other small molecules identified through GC-MS analysis, some of which have demonstrated insecticidal activity in previous research. For instance, methyl salicylate exhibited fumigant and contact toxicity against *Callosobruchus chinensis*, with an LC_50_ of 3.41 mg per L air and 28.89 μg per beetle at a 24-h exposure time [53]. Stilbene derivatives showed antifeedant activity against *Brontispa longissima* larvae, with LC_50_ values ranging from 0.218 to 2.501 mg/L [54]. Interestingly, the effects of chronic and acute exposure to phenanthrene were evaluated on the activities of acetylcholinesterase, α-esterase, and β-esterase, as well as on larval development and histological alterations of *Chironomus sancticaroli* larvae [55]. Hence, acute exposure to high concentrations of phenanthrene (1.2 mg/L) increased the activity of α-esterase and β-esterase after 48 and 72 h, while chronic exposure increased β-esterase activity at 0.12 mg/L. In addition, histological alterations were observed in the midgut, fat body, salivary glands, and Malpighian Tubules at both acute and chronic exposures, and larval development was delayed with a reduction in body length and molting [55]. Using GC-MS analysis, methyl palmitate was identified as another small molecule present in the extract. Although its insecticidal activity has not been established yet, but this compound has showed a strong acaricidal effect with 62.8 % mortality at 1 mg/mL at 24 h against *Tetranychus cinnabarinus* [56]. Recently, the larvicidal efficacy of bis-(2-ethylhexyl) phthalate was tested against *Culex quinquefasciatus* larvae, with 100 % mortality observed at 250 ppm during 72 h, and an LC_50_ value of 67.03 ppm [57]. In addition, acetylcholinesterase inhibition ranging from 29 % to 75.33 % was observed over a concentration range of 50–250 ppm. According to the analytical findings, the primary small molecule compounds within the extract comprised 6,6-dideutero-nonen-1-ol-3 (11.3 %), 4-cyclopentene-1,3-dione, 4,5-bis(4-fluorophenyl)-2-phenyl- (12.56 %), and bis(2-ethylhexyl) phthalate, with the insecticidal efficacy of the bis(2-ethylhexyl) phthalate compound being established. Consequently, given the significant concentration of this compound in the extract, a substantial portion of the insecticidal activity of the *Viola ignobilis* extract could be attributed to its presence. However, the possible effects of other small compounds, particularly those with confirmed insecticidal properties, should not be disregarded. Based on these documents, it can be concluded that the *V. ignobilis* extract, as a mixture of bioactive compounds, may provide (i) a broader spectrum of activity, potentially targeting multiple biological pathways and reducing the likelihood of resistance development, (ii) have synergistic effects, which can enhance their overall efficacy, (iii) can enable the use of lower concentrations of each individual compound, reducing the risk of environmental contamination and minimizing potential negative effects on non-target organisms, and (iv) provide a more sustainable approach to pest control, reducing the reliance on single compounds and promoting a more diverse and resilient ecosystem.

The presence of different bioactive compounds in plant extracts leads to the demonstration of different mechanisms of action and greater target specificity against insects. By comparing to the insecticidal Bt toxins, both Bt toxins and plant extracts have the ability to control pests in agriculture through bioinsecticide formulations [24]. Bt toxins are well-known insecticides that are deadly to plant pests but non-toxic to humans. Due to their mechanism of action through interaction with specific receptors in the insect [58], the possibility of insect resistance is high. The use of cyclotide-containing extracts, such as *V. ignobilis* extract, may eliminate this concern as cyclotides have the ability to directly penetrate the cell membrane and disrupt it [59]. Additionally, a combination of different small molecules in the extract can exhibit a variety of modes of action, making it difficult for insects to evade detection or utilize different suppression mechanisms. These findings indicate the important role of plant bioactive compounds in pest control and their potential for commercial production of bioinsecticides is very attractive and practical. Therefore, purification and bioassay of individual compounds in *V. ignobilis* extract will advise in future works to confirm their exact role in pest control.

## 5 Conclusion

The importance of nature-derived compounds in a safe way to control pests has provoked researchers' interest in finding novel insecticides. Due to the distinctive properties of cyclotide-containing extracts, i.e., high stability of cyclotides to chemical, thermal, and enzymatical digestion, and the diverse pesticidal behavior of a combination of various bioactive compounds, they are a valuable candidate for formulating bioinsecticides. Therefore, our findings demonstrate the insecticidal potency and effectiveness of the cyclotide-containing extract obtained from *V. ignobilis*. Furthermore, the extract exhibited no remarkable cytotoxic effects on the human control cell line HEK293 (at incubation times of up to 24 h), indicating a potential safe use as bioinsecticide. These results therefore open new avenues to develop bioinsecticides similar to the existing product Sero-X.

## CRediT authorship contribution statement

**Mohammad Sadegh Taghizadeh:** Writing – review & editing, Writing – original draft, Visualization, Validation, Software, Project administration, Methodology, Investigation, Formal analysis, Data curation, Conceptualization. **Ali Niazi:** Writing – review & editing, Validation, Supervision, Resources, Funding acquisition, Data curation. **Bernhard Retzl:** Software, Formal analysis. **Christian W. Gruber:** Writing – review & editing, Formal analysis, Resources, Supervision, Validation.

## Data and code availability statement

Data will be made available on request.

## Ethical statement

This study did not engage in any human or animal testing.

## Declaration of competing interest

The authors declare that they have no known competing financial interests or personal relationships that could have appeared to influence the work reported in this paper.

## References

[1] Hegde J.N., Ashrith K., Suma G., Chakravarthy A., Gopalkrishna H. (2020).

[2] Mehrnejad M. (2001). The current status of pistachio pests in Iran. Cah. Options Mediterr..

[3] Jalalizand A.R., Mirhendi H., Karimi A., Modaresi M., Mahmoodi E. (2012). Morphological and molecular identification aphids of Rosae. Apcbee Procedia.

[4] Goff G.L., Giraudo M. (2019). Effects of pesticides on the environment and insecticide resistance. Olfactory Concepts of Insect Control-Alternative to Insecticides, Springer.

[5] Srivastava S., Mishra A., Mishra R., Mohanty A. (2022). Biopesticidal potential of cyclotides: an insight. Phytochemistry Rev..

[6] Kumar S. (2012). Biopesticides: a need for food and environmental safety. J Biofertil Biopestic.

[7] Kumar J., Ramlal A., Mallick D., Mishra V. (2021). An overview of some biopesticides and their importance in plant protection for commercial acceptance. Plants.

[8] Liu X. (2021). Overview of mechanisms and uses of biopesticides. Int. J. Pest Manag..

[9] Taghizadeh M.S., Niazi A., Moghadam A., Afsharifar A.R. (2020). The potential application of the protein hydrolysates of three medicinal plants: cytotoxicity and functional properties. Journal of food science.

[10] Taghizadeh M.S., Niazi A., Moghadam A., Afsharifar A.R. (2021). Novel bioactive peptides of Achillea eriophora show anticancer and antioxidant activities. Bioorg. Chem..

[11] Elshafie H.S., Camele I., Mohamed A.A. (2023). A Comprehensive review on the biological, agricultural and pharmaceutical properties of secondary metabolites based-plant origin. Int. J. Mol. Sci..

[12] Yuan H. (2022). Bioactive peptides of plant origin: distribution, functionality, and evidence of benefits in food and health. Food Funct..

[13] Huang Y.-H., Du Q., Craik D.J. (2019). Cyclotides: disulfide-rich peptide toxins in plants. Toxicon.

[14] Quimbar P. (2013). High-affinity cyclic peptide matriptase inhibitors. J. Biol. Chem..

[15] Hellinger R. (2014). Immunosuppressive activity of an aqueous Viola tricolor herbal extract. J. Ethnopharmacol..

[16] Pränting M., Lööv C., Burman R., Göransson U., Andersson D.I. (2010). The cyclotide cycloviolacin O2 from Viola odorata has potent bactericidal activity against Gram-negative bacteria. Journal of antimicrobial chemotherapy.

[17] Burman R. (2011). Cytotoxic potency of small macrocyclic knot proteins: structure–activity and mechanistic studies of native and chemically modified cyclotides. Org. Biomol. Chem..

[18] Huang Y.-H., Colgrave M.L., Clark R.J., Kotze A.C., Craik D.J. (2010). Lysine-scanning mutagenesis reveals an amendable face of the cyclotide kalata B1 for the optimization of nematocidal activity. J. Biol. Chem..

[19] Plan M.R.R., Saska I., Cagauan A.G., Craik D.J. (2008). Backbone cyclised peptides from plants show molluscicidal activity against the rice pest Pomacea canaliculata (golden apple snail). J. Agric. Food Chem..

[20] Jennings C., West J., Waine C., Craik D., Anderson M. (2001). Biosynthesis and insecticidal properties of plant cyclotides: the cyclic knotted proteins from Oldenlandia affinis. Proc. Natl. Acad. Sci. USA.

[21] Riaz M. (2023). Phytobioactive compounds as therapeutic agents for human diseases: a review. Food Sci. Nutr..

[22] Moghadam A. (2023). System network analysis of Rosmarinus officinalis transcriptome and metabolome—key genes in biosynthesis of secondary metabolites. PLoS One.

[23] Moghadam A. (2024). Exploring novel insights: methyl jasmonate treatment reveals novel lncRNA-mediated regulation of secondary metabolite biosynthesis pathways in Echinacea purpurea. Food Biosci..

[24] Oguis G.K., Gilding E.K., Jackson M.A., Craik D.J. (2019). Butterfly pea (Clitoria ternatea), a cyclotide-bearing plant with applications in agriculture and medicine. Frontiers in plant science.

[25] Colgrave M.L. (2008). Cyclotides: natural, circular plant peptides that possess significant activity against gastrointestinal nematode parasites of sheep. Biochemistry.

[26] Quadros D.G., Johnson T.L., Whitney T.R., Oliver J.D., Oliva Chávez A.S. (2020). Plant-derived natural compounds for tick pest control in livestock and wildlife: pragmatism or utopia?. Insects.

[27] Aslam L., Kaur R., Kapoor N., Mahajan R. (2020). Phytochemical composition and antioxidant activities of leaf extracts of Viola odorata from Kishtwar, Jammu and Kashmir. J. Herbs, Spices, Med. Plants.

[28] Feyzabadi Z., Ghorbani F., Vazani Y., Zarshenas M.M. (2017). A critical review on phytochemistry, pharmacology of Viola odorata L. and related multipotential products in traditional Persian medicine. Phytother Res..

[29] Farhadpour M. (2016). Microwave-assisted extraction of cyclotides from Viola ignobilis. Anal. Biochem..

[30] Hashempour H., Koehbach J., Daly N.L., Ghassempour A., Gruber C.W. (2013). Characterizing circular peptides in mixtures: sequence fragment assembly of cyclotides from a violet plant by MALDI-TOF/TOF mass spectrometry. Amino acids.

[31] Esmaeili M.A. (2016). Viola plant cyclotide vigno 5 induces mitochondria-mediated apoptosis via cytochrome C release and caspases activation in cervical cancer cells. Fitoterapia.

[32] Taghizadeh M.S. (2022). Discovery of the cyclotide caripe 11 as a ligand of the cholecystokinin-2 receptor. Sci. Rep..

[33] Wang C.K., Kaas Q., Chiche L., Craik D.J. (2007). CyBase: a database of cyclic protein sequences and structures, with applications in protein discovery and engineering. Nucleic acids research.

[34] Shahraki Z., Taghizadeh M.S., Niazi A., Rowshan V., Moghadam A. (2024). Enhancing bioactive compound production in Salvia mirzayanii through elicitor application: insights from in vitro and in silico studies. Food Biosci..

[35] Ahmed M. (2020). Insecticidal activity and biochemical composition of Citrullus colocynthis, Cannabis indica and Artemisia argyi extracts against cabbage aphid (Brevicoryne brassicae L.). Sci. Rep..

[36] Abbott W.S. (1925). A method of computing the effectiveness of an insecticide. J. Econ. Entomol..

[37] Sadeghi A., Van Damme E.J., Smagghe G., Cullen E. (2009). Evaluation of the susceptibility of the pea aphid, Acyrthosiphon pisum, to a selection of novel biorational insecticides using an artificial diet. Journal of Insect science.

[38] Omer A., Granett J., Karban R., Villa E. (2001). Chemically-induced resistance against multiple pests in cotton. Int. J. Pest Manag..

[39] Pascual-Villalobos M., Robledo A. (1998). Screening for anti-insect activity in Mediterranean plants. Industrial crops and products.

[40] Taghizadeh M.S., Niazi A., Moghadam A., Afsharifar A. (2022). Experimental, molecular docking and molecular dynamic studies of natural products targeting overexpressed receptors in breast cancer. PLoS One.

[41] De Geyter E., Smagghe G., Rahbé Y., Geelen D. (2012). Triterpene saponins of Quillaja saponaria show strong aphicidal and deterrent activity against the pea aphid Acyrthosiphon pisum. Pest Manag. Sci..

[42] Sun Z. (2023). Matrine can be absorbed and transmitted bidirectionally to defend aphids (Hemiptera: Aphididae) on wheat and pepper. Pest Manag. Sci..

[43] Bemani M., Moravvej G., Izadi H., Sadeghi-Namaghi H. (2021). Detoxifying enzyme activities in the common pistachio psylla and the coccinellid predator. J. Agric. Sci. Technol..

[44] Noureldeen A. (2022). Aphicidal activity of five plant extracts applied singly or in combination with entomopathogenic bacteria, Xenorhabdus budapestensis against rose aphid, Macrosiphum rosae (Hemiptera: Aphididae). J. King Saud Univ. Sci..

[45] Mensah R., Leach D., Young A., Watts N., Glennie P. (2015). Development of C litoria ternatea as a biopesticide for cotton pest management: assessment of product effect on H elicoverpa spp. and their natural enemies. Entomol. Exp. Appl..

[46] Tembo Y. (2018). Pesticidal plant extracts improve yield and reduce insect pests on legume crops without harming beneficial arthropods. Front. Plant Sci..

[47] Amirzade N., Izadi H., Jalali M.A., Zohdi H. (2014). Evaluation of three neonicotinoid insecticides against the common pistachio psylla, Agonoscena pistaciae, and its natural enemies. J. Insect Sci..

[48] Dancewicz K. (2020). Behavioral and physiological effects of Viola spp. cyclotides on Myzus persicae (Sulz.). J. Insect Physiol..

[49] Oguis G.K. (2020). Insecticidal diversity of butterfly pea (Clitoria ternatea) accessions. Ind. Crop. Prod..

[50] Broussalis A.M., Clemente S., Ferraro G.E. (2010). Hybanthus parviflorus (Violaceae): insecticidal activity of a south American plant. Crop Protect..

[51] Pinto M.F. (2012). Identification and structural characterization of novel cyclotide with activity against an insect pest of sugar cane. J. Biol. Chem..

[52] Matsuura H.N., Poth A.G., Yendo A.C., Fett-Neto A.G., Craik D.J. (2016). Isolation and characterization of cyclotides from Brazilian Psychotria: significance in plant defense and co-occurrence with antioxidant alkaloids. Journal of natural products.

[53] Park C.G., Shin E., Kim J. (2016). Insecticidal activities of essential oils, Gaultheria fragrantissima and Illicium verum, their components and analogs against Callosobruchus chinensis adults. J. Asia Pac. Entomol..

[54] Liu Y.-Q. (2013). Synthesis and insect antifeedant activity of stilbene derivatives against Brontispa longissima larvae. Med. Chem. Res..

[55] Richardi V. (2018). Effects of phenanthrene on different levels of biological organization in larvae of the sediment-dwelling invertebrate Chironomus sancticaroli (Diptera: chironomidae). Environmental pollution.

[56] Wang Y. (2009). Methyl palmitate, an acaricidal compound occurring in green walnut husks. J. Econ. Entomol..

[57] Javed M.R. (2022). The antibacterial and larvicidal potential of bis-(2-ethylhexyl) phthalate from lactiplantibacillus plantarum. Molecules.

[58] Melo A.L.d.A., Soccol V.T., Soccol C.R. (2016). Bacillus thuringiensis: mechanism of action, resistance, and new applications: a review. Crit. Rev. Biotechnol..

[59] Barbeta B.L., Marshall A.T., Gillon A.D., Craik D.J., Anderson M.A. (2008). Plant cyclotides disrupt epithelial cells in the midgut of lepidopteran larvae. Proc. Natl. Acad. Sci. USA.

